# Optical Coherence Tomography in Mild Cognitive Impairment: A Systematic Review and Meta-Analysis

**DOI:** 10.3389/fneur.2020.578698

**Published:** 2020-10-16

**Authors:** Alvaro J. Mejia-Vergara, Paula Restrepo-Jimenez, Victoria S. Pelak

**Affiliations:** ^1^Department of Neuro-Ophthalmology, Stein and Doheny Eye Institutes, University of California, Los Angeles, Pasadena, CA, United States; ^2^Ophthalmology Department, San Ignacio University Hospital, Pontificia Universidad Javeriana School of Medicine, Bogotá, Colombia; ^3^Departments of Neurology and Ophthalmology, University of Colorado School of Medicine, Aurora, CO, United States

**Keywords:** Alzheimer's disease, mild cognitive impairment, optical coherence tomography, retina, biomarker, systematic review, meta–analysis

## Abstract

**Purpose:** The use of optical coherence tomography (OCT) of the retina to detect inner retinal degeneration is being investigated as a potential biomarker for mild cognitive impairment (MCI) and Alzheimer's disease (AD), and an overwhelming body of evidence indicates that discovery of disease-modifying treatments for AD should be aimed at the pre-dementia clinical stage of AD, i.e., MCI. We aimed to perform a systematic review and meta-analysis on retinal OCT in MCI.

**Methods:** We performed a systematic review of the English literature in three databases (PubMed, Embase, and Latindex) for studies that measured retinal thickness using OCT in people with MCI and healthy controls, age 50 or older, between 1 January 2000 and 31 July 2019. Only cohort and case-control studies were reviewed, and independent extraction of quality data and established objective data was performed. We calculated the effect size for studies in the review that met the following criteria: (1) a statistically significant difference between MCI subjects and normal controls for several OCT variables, (2) use of spectral domain OCT, and (3) use of APOSTEL recommendations for OCT reporting. Weighted Hedges' g statistic was used to calculate the pooled effect size for four variables: *ganglion cell layer-inner plexiform layer* (*GCL-IPL) complex thickness* in micrometers (μm), *circumpapillary retinal nerve fiber layer (pRNFL) thickness* in μm, *macular thickness* in μm, and *macular volume* in μm^3^. For variables with high heterogeneity, a multivariate meta-regression was performed. We followed the PRISMA guidelines for systematic reviews.

**Results:** Fifteen articles met the inclusion criteria. A total of 58.9% of MCI patients had statistically significant thinning of the pRNFL compared with normal subjects, while 61.6% of all MCI patients who had macular volume measured had a statistically significant reduction in volume compared with controls, and 50.0% of the macular GCL-IPL complexes measured demonstrated significant thinning in MCI compared with normal controls. Meta-analysis demonstrated a large effect size for decreased macular thickness in MCI subjects compared with normal controls, but there was a substantial heterogeneity for macular thickness results. The other variables did not demonstrate a significant difference and also had substantial heterogeneity. Meta-regression analysis did not reveal an explanation for the heterogeneity.

**Conclusions:** A better understanding of the cause of retina degeneration and longitudinal, standardized studies are needed to determine if optical coherence tomography can be used as a biomarker for mild cognitive impairment due to Alzheimer's disease.

## Introduction

Optical coherence tomography (OCT) of the retina has been proposed as a potential biomarker for Alzheimer's disease (AD), because of the known loss of retinal ganglion cells, and their axons in the inner retina found on histopathological studies of AD autopsy retinal samples and OCT studies revealing inner retinal thinning in people with a clinical diagnosis of AD compared with non-demented, age-matched controls (1–4). OCT is a non-invasive imaging technique that gives a cross-sectional, detailed image of the retina and its histological components (5). OCT technology can provide information about the thickness of the individual layers of the retina, as well as their spatial relationship within the retina. Changes in these measurements and relationships are well-described for many other ophthalmological and neuro-ophthalmological entities, and many patterns are recognizable for the trained physician (6–11). Changes in these OCT patterns have been studied in patients with a clinical diagnosis of AD diagnosis but very little data is available for the early stages of the disease (1–4). Converging lines of evidence indicate that for therapeutic interventions to have a significant clinical impact on the Alzheimer's disease, treatments should be aimed at the pre-dementia stage, referred to as mild cognitive impairment (MCI), and the pre-symptomatic stage of AD, and biomarkers for these stages of disease will be a key to successful development of disease-modifying therapies (12–16).

A previous systematic review regarding OCT and mild cognitive impairment was published in 2016 (17); however, recently published data and technical advances in OCT hardware and software analysis highlights the importance of updating and summarizing recent evidence. The main objective of this systematic review and meta-analysis is to report existing evidence available in the literature regarding retinal OCT findings in the clinical, pre-dementia stage of AD in people older than age 50 years compared with healthy individuals, with attention to retinal scanning location (macula vs. circumpapillary regions) and the reported retina layers [ganglion cell layer (GCL) vs. retinal nerve fiber layer (RNFL)]. To this end, the systematic review and meta-analysis aims to answer the following questions:

- Are there differences between MCI and controls in: (1) circumpapillary RNFL thickness and (2) macular volume, macular total thickness, or macular GCL-inner plexiform layer (IPL) thickness?
- Do study findings depend on OCT location (macula or circumpapillary)?

## Evidence Acquisition

### Protocol and Registration

In accordance with the PRISMA guidelines (18), this systematic review was registered with the International Prospective Register of Systematic Reviews (PROSPERO) as a protocol with registration identification CRD42018110842.

### Eligibility Criteria

Randomized trials, clinical control trials, cohort, and case-control trials that measured retinal parameters using optical coherence tomography and included participants with mild cognitive impairment and healthy controls age 50 or older were included. Studies reporting OCT (all modalities) in people with mild cognitive impairment were considered for inclusion.

### Information Sources and Search

Authors AM-V and VP conducted the search for articles published between 1 January 2000 and 31 July 2019. The terms included were: “mild cognitive impairment” AND “optical coherence tomography.” Electronic databases addressed were PubMed, Latindex, and Embase®. The search was limited by language and date as only studies published in English and between January 1st, 2000 and 31st July 2019. The following was the search criteria: “mild cognitive impairment” [All Fields] AND “optical coherence tomography” [All Fields] AND [(“2000/01/01” [PDAT]: “2019/07/31” [PDAT]) AND English [lang]].

### Study Selection

Two authors, AM-V and VP, independently screened the studies for inclusion by title and abstract assessment. In case of doubt, full-text reports were obtained to further clarify the inclusion of studies. If disagreements occurred, they were resolved by consensus.

### Data Collection Process and Data Items

The studies yielded in the database search were uploaded to an Excel spreadsheet and managed in the electronic database administrator Mendeley. Data regarding mild cognitive impairment participants and controls (number of participants, age, gender, methods for diagnosis, and diagnostic criteria for mild cognitive impairment), OCT techniques (spectral domain or time domain), scan location of OCT (macula, circumpapillary, or both), segmentation technique (manual, automated, or both), and available retinal measurements (macular thickness, macular volume, retinal nerve fiber layer thickness, retinal ganglion cell thickness, inner retinal layers, outer retinal layers) were retrieved by the reviewers and stored in an Excel spreadsheet for further analysis.

### Risk of Bias

The Robvis tool (RRID pending) was used to evaluate the risk of bias in each article (19). Additionally, quality assessment was performed through a critical appraisal of included studies. The following standards were considered:

- Diagnostic criteria used for MCI and method for assessing MCI and controls.
- Collection of data (retrospective or prospective).
- Cross-section vs. longitudinal data with follow-up.
- Blinding of both participants and study personnel to groups.
- Use of Advised Protocol for OCT Study Terminology and Elements (APOSTEL) recommendations for reporting OCT results (20).

### Summary and Analysis

Studies were summarized in a narrative way according to each of the three principal questions aimed by this review by the three authors. Tables 1–3 were used to summarize and describe the characteristics of the included studies as well as the main findings of each.

**Table 1:** General description from the 15 studies meeting inclusion criteria.

**Study**	**Location**	**Diagnostic criteria**	**Main findings**
Paquet et al. (21)	France	NINCDS-ADRDA (22)	MCI showed thinner average pRNFL thickness compared with normal controls.
Kesler et al. (23)	Israel	Petersen Criteria (24)	MCI showed significantly thinner average pRNFL thickness compared with controls and a significant difference in the inferior quadrant thickness.
Shen et al. (25)	China	Petersen Criteria (24)	MCI showed significantly thinner inferior and nasal pRNFL quadrants but no difference in average pRNFL compared with controls. No significant difference in macula central thickness and macular volume thickness found between MCI and controls.
Ascaso et al. (26)	Spain	Winbald (27)	MCI showed a significant reduction in average pRNFL thickness and in all quadrants compared with age-matched controls.
Oktem et al. (28)	Turkey	Not mentioned	MCI showed significantly reduced average pRNFL thickness compared with the controls.
Liu et al. (29)	China	Petersen Criteria (24)	MCI showed significantly reduced average pRNFL and superior quadrant thickness compared with controls.
Gao et al. (30)	China	Petersen Criteria (24)	MCI had significantly decreased macular volume when adjusted for age and sex compared with controls. No significant difference was noted for the overall pRNFL.
Cheung et al. (31)	Singapore	Petersen Criteria (24)	MCI had significantly reduced mGCL-IPL thickness in all but the superior temporal sector compared with healthy controls. pRNFL thickness measures were not different between MCI compared with healthy controls.
Pillai et al. (32)	USA	NIA-AA (33)	MCI and controls had no significant differences in average pRNFL thickness, macular volume, or mGCL-IPL thickness.
Giménez Castejón et al. (34)	Spain	Neurological examination and DSM-IV (35)	MCI had significantly decreased total macular thickness compared with controls.
Ferrari et al. (36)	Italy	NIA-AA (33)	MCI had significant thinning of the average pRNFL compared with controls. There was no difference in pGCL-IPL thickness.
Uchida et al. (37)	USA	NIA-AA (33)	MCI showed no significant differences between MCI and controls in measurements of macular outer layers or the total macular thickness.
Shao et al. (38)	USA	NIA-AA (33)	MCI had significantly decreased total macular thickness and mGCL-IPL thickness in the superior and nasal quadrants compared with controls.
Lad et al. (39)	USA	NINCDS-ADRD (22)	MCI had no significant differences in average mRNFL thickness, mGCL-IPL compared with controls.
Almeida et al. (40)	Brazil	Collie (41)	MCI had no significant difference in pRNFL thickness compared with controls. Total macular thickness was decreased in MCI vs. control.

*MCI, mild cognitive impairment; RNFL, retinal nerve fiber layer; AD Alzheimer's disease; GCL-IPL, ganglion cell-inner plexiform layer; NINCDS-ADRD, National Institute of Neurological Disorders and Stroke-Alzheimer Disease and Related Disorders Association; NIA-AA, National Institutes of Health-Alzheimer's Association*.

*Results of this study are reported as the change in cell layers, not individual values are reported*.

**Table 2:** OCT retinal thickness measures from the 15 studies meeting inclusion criteria.

**Study**	**Subjects, n**		**Age, mean (SD)**		**Gender, Female (%)**		**Notes**	**Retinal scan location**	**OCT technique**	**Macular thickness μm, mean (SD) MCI**	**Macular thickness μm, mean (SD) NC**	**Macular volume μm, mean (SD) MCI**	**Macular volume μm, mean (SD) NC**	**pRNFL thickness μm, mean (SD) MCI**	**pRNFL thickness μm, mean (SD) NC**	**GC-IPL thickness μm, mean (SD) MCI**	**GC-IPL thickness μm, mean (SD) NC**	**mRNFL thickness in μm, mean (SD) MCI**	**mRNFL thickness in μm, mean (SD) NC**	**Outer Retinal Layers μm, mean (SD) MCI**	**Outer Retinal Layers μm, mean (SD) NC**
	**MCI**	**NC**	**MCI**	**NC**	**MCI**	**NC**															
Paquet et al. (21)	23	15	78.7 (6.2)	75.5 (5.1)	65.2	86.6	NA	CP	TD	ND	ND	ND	ND	89.3 (2.7)^*^	102.2 (1.8)	ND	ND	ND	ND	ND	ND
Kesler et al. (23)	24	24	71.0 (10.0)	70.9 (9.2)	NR	NR	NA	CP	TD	ND	ND	ND	ND	85.8 (10.0)^*^	94.3 (11.3)	ND	ND	ND	ND	ND	ND
Shen et al. (25)	23	52	74.1	74.1	43.5	59.6	NA	B	TD	VNR	VNR	VNR	VNR	Average 82.6 (10.5); Inferior: 104.5 (17.6)^*^; nasal 61.5 (8.1)^*^	Average 85.6 (10.2); inferior 109.3 (21.3); nasal 64.8 (8.4)	ND	ND	ND	ND	ND	NR
Ascaso et al. (26)	21	41	NR	72.9	NR	51,2	NA	B	TD	OD: 228.19 (24.63)	OD: 208.66 (24.70)	OD: 7.06 (0.15)	OD: 6.51 (0.40)	OD: 86.03 (7.26)^*^	OD: 103.57 (8.94)	ND	ND	ND	ND	ND	ND
										OS: 224.24 (23.34)	OS: 209.96 (22.04)	OS: 6.97 (0.48)	OS: 6.59 (0.38)	OS: 87.28 (7.22) ^*^	OS: 102.65 (6.89)						
Oktem et al. (28)	35	35	74.1 (6.3)	70.2 (8.0)	57.1	65.7	NA	CP	SD	ND	ND	ND	ND	82.5 (7.3)^*^	91.5 (7.1)	ND	ND	ND	ND	ND	ND
Liu D et al. (29)	26	39	71.3 (4.9)	69.7 (7.8)	62.5	56.4	NA	CP	TD	ND	ND	ND	ND	95.37 (17.11)^*^	100.12 (15.01)	ND	ND	ND	ND	ND	ND
Gao et al. (30)	26	21	73.42 (1.54)	72.05 (1.02)	38.5	66.6	NA	B	SD	ND	ND	9.63 (0.08) ^*^	9.96 (0.06)	92.38 (1.94)	98.60 (1.67)	ND	ND	ND	ND	ND	ND
Cheung et al. (31)	41	123	70.4	65.7 (3.77)	68.3	45.5	NA	B	SD	ND	ND	ND	ND	89.21	90.37 (1.71)	73.73 (1.35) ^*^	77.79 (1.31)	ND	ND	ND	ND
Pillai et al. (32)	21	34	68.2 (6.7)	65.1 (8.3)	57	59	Brain Atrophy Measured	B	SD	ND	ND	9.9 (0.1)	9.8 (0.1)	89.9 (2.1)	85.3 (1.6)	78.6 (1.8)	73.5 (1.4)	ND	ND	ND	ND
Giménez Castejón et al. (34)	33	25	68.74 (8.00)	68.68 (8.21)	45.5	60	NA	M	SD	259.48 (22.39) ^*^	274.96 (17.61)	ND	ND	ND	ND	ND	ND	ND	ND	ND	ND
Ferrari et al. (36)	29	49	70.45 (5.51)	68.32 (6.96)	51.7	53	GCL-IPL measured by M+A segmentation (in-house designed software)	B	SD	ND	ND	ND	ND	92.79 (10.31) ^*^	97.49 (8.52)	55.61 (8.17)	58.18 (7.94)	ND	ND	ND	ND
Uchida et al. (37)	22	36	68.9 (6.8)	65.1 (8.3)	64	61	M+A segmentation	M	SD	202.9 (3.9)	207.3 (4.2)	9.8 (0.1)	9.8 (0.1)	ND	ND	ND	ND	ND	ND	174.3 (3.1) [124.2 (3.0) ONL-EZ + 48.7 (1.4) EZ-REP]	180.2 (2.8) [131.0 (2.9) ONL-EZ + 49.2 (1.3) EZ-RPE]
Shao et al. (38)	24	21	69 (8)	68 (7)	41.6	52.3	NA	M	SD	VNR, but significant^*^	VNR	ND	ND	ND	ND	76^*^	79	ND	ND	ND	ND
Lad et al. (39)	15	18	73.07	75.17	5.3	4.4	M+A segmentation	B	SD	ND	ND	ND	ND	95.69 (10.92)	97.15 (8.94)	63.71 (7.12)	64.08 (3.60)	ND	ND	ND	ND
Almeida et al. (40)	23	24	67.43	64.58	82.6	66.6	NA	B	SS	268.9^*^ (3.08)	275.73 (2.26)	ND	ND	103.50 (2.42)	103.77 (2.00)	62.78 (0.95)	64.67 (0.83)	ND	ND	ND	ND

*Retinal scan location: macula (M), circumpapillary (CP), and both (B); OCT technique: spectral domain (SD), time domain (TD), and source sweep (SS)*.

*RNFL, retinal nerve fiber layer; M+A segmentation, manual and automated segmentation; GCL-IPL, retinal ganglion cell layer; VNR, measured, but value not reported; ND, not done; HA, hippocampal atrophy*.

*Results of this study are reported as the change in layers, and no individual values are reported*.

* *Statistically significant difference*.

**Table 3:** Quality assessment of the 15 studies meeting inclusion criteria.

**Study**	**Diagnostic criteria**	**Biomarkers (MCI)**	**Biomarkers (NC)**	**Collection of data**	**Cross-sectional/longitudinal data**	**Follow-up**	**Double-blind**	**Use of APOSTEL**
Paquet et al. (21)	NINCDS-ADRDA (22)	No	No	Prospective	Cross-sectional	No	No	No
Kesler et al. (23)	Petersen Criteria (24)	No	No	Prospective	Cross-sectional	No	No	No
Shen et al. (25)	Petersen Criteria (24)	No	No	Prospective	Cross-sectional	No	No	No
Ascaso et al. (26)	Winbald (27)	No	No	Prospective	Cross-sectional	No	No	No
Oktem et al. (28)	Not mentioned	No	No	Prospective	Cross-sectional	No	No	No
Liu D et al. (29)	Petersen Criteria (24)	No	No	Prospective	Cross-sectional	No	No	No
Gao et al. (30)	Petersen Criteria (24)	No	No	Prospective	Cross-sectional	No	No	No
Cheung et al. (31)	Petersen Criteria (24)	No	No	Prospective	Cross-sectional	No	No	No
Pillai et al. (32)	NIA-AA (33)	Hippocampal atrophy	Non-hippocampal atrophy	Prospective	Cross-sectional	No	No	Yes
Giménez Castejón et al. (34)	Neurological examination and DSM-IV (35)	No	No	Retrospective	Cross-sectional	No	No	No
Ferrari et al. (36)	NIA-AA (33)	No	No	Prospective	Cross-sectional	No	No	Yes
Uchida et al. (37)	NIA-AA (33)	No	No	Prospective	Cross-sectional	No	No	Yes
Shao et al. (38)	NIA-AA (33)	No	No	Prospective	Cross-sectional	No	No	Yes
Lad et al. (39)	NINCDS-ADRD (22)	No	No	Prospective	Cross-sectional	No	No	Yes
Almeida et al. (40)	NINCDS-ADRDA (22)	No	No	Prospective	Cross-sectional	No	No	Yes

*MCI, mild cognitive impairment; NC, normal controls; MRI, magnetic resonance imaging; NINCDS-ADRD, National Institute of Neurological and Communicative Disorders and Stroke-Alzheimer Disease and Related Disorders Association; NIA-AA, National Institutes of Health-Alzheimer's Association*.

*Results of this study are reported as the change in layers, and no individual values were reported*.

### Meta-Analysis

We calculated the effect size for studies in the review that met the following criteria: (1) a statistically significant difference between MCI subjects and normal controls for several OCT variables, (2) use of spectral domain OCT, and (3) APOSTEL recommendations for OCT reporting. Criteria-specified spectral domain technology was chosen because it is currently the most commonly used OCT technology, and APOSTEL reporting ensures a standardized approach. We excluded Shao et al. study (31) because the authors did not report standard deviations for their dataset or an exact *p*-value. Statistical analysis was performed with the Stata data analysis software v.14.0 (StataCorp 2015, USA). The Hedges' g test, or biased-corrected sample, was used to calculate the effect sizes, because the sample sizes for all studies were small. Random effects model was used to pool the effect sizes. Four different variables were analyzed: *GCL-IPL complex thickness* in micrometers (μm), *pRNFL thickness* in μm, *macular thickness* in μm, and *macular volume* in μm^3^. For variables with high heterogeneity, a multivariate meta-regression was performed.

## Results

**Figure 1:**
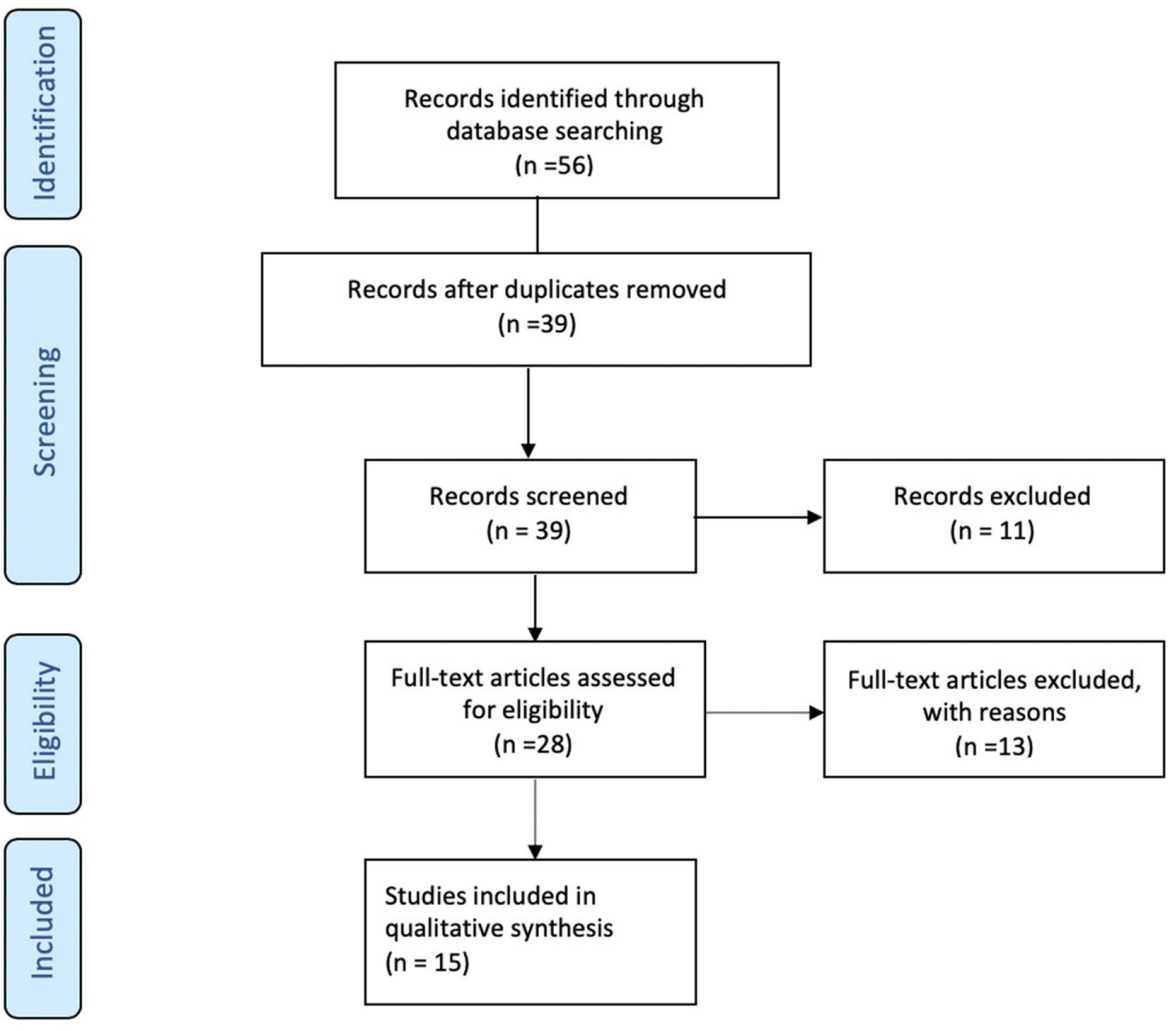
Flow Diagram. PRISMA flow-diagram of electronic database search in Pubmed, Embase, and Latindex. The diagram shows the systematic search of the Pubmed, Embase, and Latindex electronic databases of publications from January 2000 to July 2019. The Preferred Reporting Items for Systematic Reviews and Meta-Analyses Protocols (PRISMA-P) guidelines (18) were used to assess the current state of knowledge regarding pre-dementia stage of Alzheimer's disease, Mild Cognitive Impairment (or MCI) and retinal measures by optical coherence tomography.

**Figure 2:**
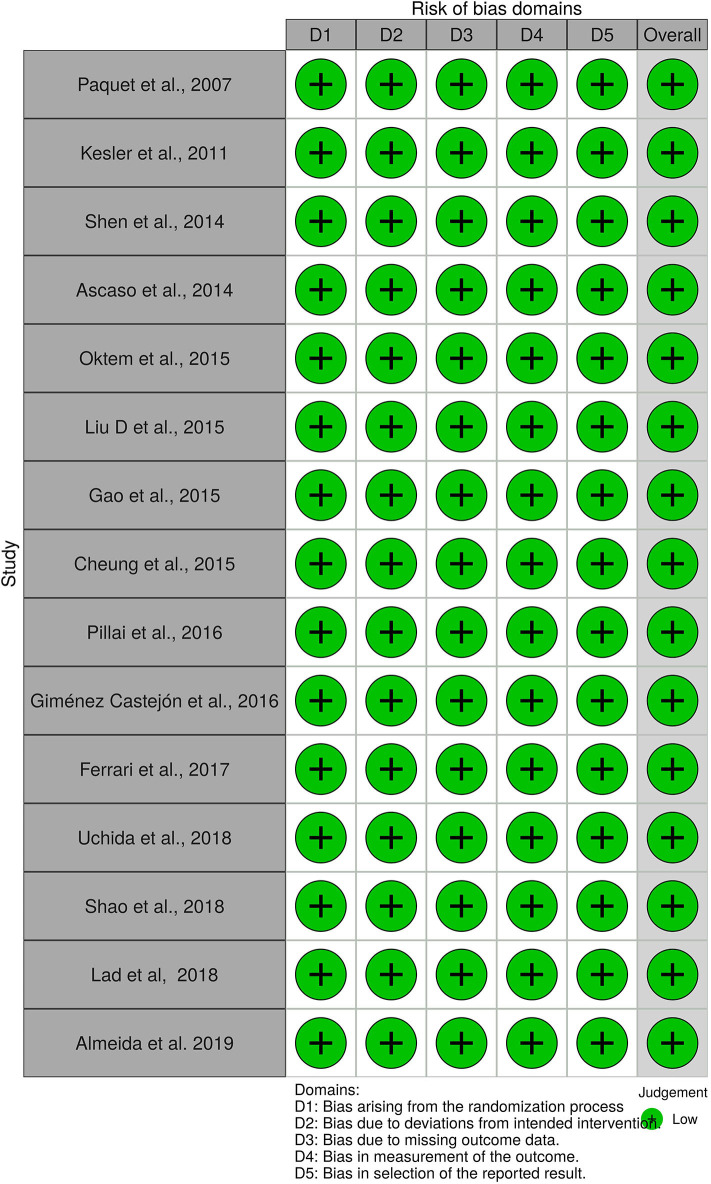
Risk of bias assesment with the ROBVIS tool of all 15 articles.

A total of 56 studies were identified through MedLine, Embase, and Latindex database search. After adjusting for duplicate data, 39 studies remained. Eleven studies were excluded because they did not meet the inclusion criteria. The remaining 28 studies were assessed in full text for more detail. Further evaluation of full texts led to the exclusion of 11 more studies because they did not meet the inclusion criteria. Two articles were excluded because the anatomical position of the OCT scans was not clearly noted. Communication with the authors was attempted, but no clarification was possible (42, 43). Finally, a total of 15 studies meeting inclusion criteria were evaluated and summarized (21, 23, 25, 26, 28–32, 34, 36–40) (see Figure 1). The general description and main findings of the included studies are described in Table 1. A detailed description of the relevant outcomes is provided in Table 2 and a description of quality assessment items that were evaluated for the included studies is provided in Table 3. Risk of bias results using the Robvis tool was overall low and is presented in Figure 2.

### Demographics, Cohorts, and Study Design

The countries with the highest number of publications were from the USA (4/15) and China (3/15) (25, 29, 30, 32, 37–39). Other studies belonged to countries in Asia, Europe, and South America (21, 23, 26, 28, 31, 34, 36, 40). Overall, there were seven different diagnostic criteria used to define MCI and one study did not report the diagnostic criteria. The Petersen criteria for MCI appeared to be the most frequently used (5/15), followed by the National Institute of Neurological Disorders and Communicative Disorders and Stroke-Alzheimer Disease and Related Disorders Association (NINCDS-ADRDA) criteria (3/15) (22, 24, 44). Four studies used the current National Institute of Aging-Alzheimer's Association (NIA-AA) criteria, two used the DSM-IV criteria, and two studies used unconventional diagnostic criteria that are not interchangeable with the most commonly used criteria for MCI. See Table 2 for details. All studies reported the number of cases and controls; however, significant heterogeneity was observed in the sample size and proportion between groups. The majority of studies reported a greater frequency of females among their participants. No statistical differences related to gender were reported by the authors of the studies. Similarly, none of the included studies reported statistically significant differences between groups regarding mean age. Among cases, the lowest mean age was reported by Pillai et al. (32) at 68.2 years, and the highest age reported was by Paquet et al. (21) at 79 years. For all of the controls, the age range was between 65 and 75.5 years (32, 37). Only one study reported the use of biomarkers: Pillai et al. (32) measured hippocampal atrophy (a non-specific biomarker of neurodegeneration) in both groups. Thus, amyloid and tau markers were not reported for any of the studies included here.

### OCT Methods

Reporting of the retina scan location (macular vs. circumpapillary) varied, as did the specific measures of retinal thickness evaluated. The most common report was both circumpapillary and macular scanning (approximately 46.6%), followed by circumpapillary scan alone (33.3%), and macular scan alone (20%). Macular thickness was reported by five studies (25, 26, 34, 37, 40). Macular volume was reported in five studies with some studies reporting both volume and thickness (25, 26, 30, 32, 37). Twelve studies reported circumpapillary retinal nerve fiber layer (pRNFL) measures, five reported GCL-IPL measures, and one reported an outer retinal layer measure (21, 23, 25, 26, 28–32, 38, 39). Spectral-domain OCT was the most commonly used technology (10 of 15 studies), and the remainder used time-domain OCT, Almeida et al., who used frequency domain with source sweep domain (21, 23, 25, 26, 29, 40). All of the studies used the automated segmentation technique, with Uchida et al. and Lad et al. also using manual segmentation as a “safety check.” Ferrari et al. used an in-house-developed segmentation software for GCL-IPL complex analysis from circumpapillary scans, which is a relatively novel approach (36). As noted, the quantitative data are described in Table 2 for each study. Most of the studies did not use the APOSTEL recommendations for OCT reporting, published in 2016, but more recent OCT MCI studies included in this review were more likely to adhere to APOSTEL reporting recommendations (20).

### OCT Results

Overall, 12 of the 15 studies found statistically significant retinal thinning of at least one: pRNFL, and/or the macular GCL-IPL complex, and/or the macular thickness or macular volume in subjects with MCI compared with cognitively healthy controls (total subjects MCI:controls, 328–451) (21, 23, 25, 26, 28–31, 34, 36, 38, 40).

### Circumpapillary OCT Results

Of the 12 studies that measured pRNFL (subjects MCI:controls, 307:451) (21, 23, 25, 26, 28–32, 36, 39, 40), 7 demonstrated significant pRNFL thinning in MCI compared with controls for either average pRNFL (4 of 12) or localized thinning in the superior (1 of 12) or inferior quadrants (2 of 12) or both (subjects MCI:controls, 181:255) (21, 23, 25, 26, 28, 29, 36). All studies that used time-domain OCT to measure pRNFL showed a significant difference while only two of six studies using spectral domain revealed a significant difference in pRNFL (28, 36). The five studies that found no difference in pRNFL between MCI and controls had a total of 126 MCI subjects and 196 controls (30–32, 39, 40). In summary, 58.9% of the MCI subjects with measurement of pRNFL had significant thinning compared with controls.

### Macular OCT Results

Five of the 15 studies measured the GCL-IPL complex thickness using macular OCT (subjects MCI:controls, 130:245) (32, 36, 38, 39). Two of the five studies reported significant thinning of the GCL-IPL complex in MCI patients compared with controls (subjects MCI:controls, 65:144), and both studies used spectral-domain OCT (31, 38). The other three studies that showed no significant difference between the two groups (subjects MCI:Controls, 65:101) all used spectral domain (32, 36, 39). In summary, 50.0% of the macular GCL-IPL complexes measured demonstrated significant thinning in MCI compared with controls.

Seven of the 15 studies evaluated macular volumes (subjects MCI:controls, 172:213) (25, 30, 32, 34, 37, 38, 40). In four of the seven studies, a statistically significant reduced macular volume was noted for MCI compared with controls (subjects MCI:controls, 106:91) (30, 34, 38), while three studies did not find differences (subjects MCI:controls, 66:122) (25, 32, 37). Thus, 61.6% of all MCI patients that had their macular volume measured had a statistically significant reduction in volume compared with controls.

### Studies With Macular and Circumpapillary OCT Results

Seven of the 15 studies scanned both the macula and the circumpapillary region (subjects MCI:controls, 178:321) (25, 30–32, 36, 39, 40). Five of the seven studies showed significant differences in either pRNFL or macular measures (both GCL-IPL complex or macular volume) for MCI compared with controls, but none showed both areas as significantly different from controls. Two of the five studies showed pRNFL thinning, but no changes in the GCL-IPL complex or the macular volume (subjects MCI:controls, 52:101) (25, 36). In the three other studies revealing significant differences, the authors observed significant thinning of macular structures in MCI patients compared with controls, but no significant differences in the pRNFL (subjects MCI:Controls, 90:168) (30, 31, 40). The final two studies did not find any significant changes in either pRNFL of macular structures between MCI patients and control pRNFL (subjects MCI:controls, 36:52) (39, 45). The specifics of each study were mentioned in the previous section, and details are found in Table 2.

In summary, three studies from the 15 included in this review revealed no significant differences between MCI and control in any location of the retina (total subjects MCI:controls, 58:88) (32, 37, 39). Two of these studies did not find significant differences in the pRNFL, macula CGL-ILP thickness, or macular volume compared with controls (total subjects MCI:controls, 36:52) (32, 39). One study failed to demonstrate a significant difference between macular thickness or macular volume in MCI patients compared with controls (subjects MCI:controls, 22:36) (37).

### Meta-Analysis Results

**Figure 3:**
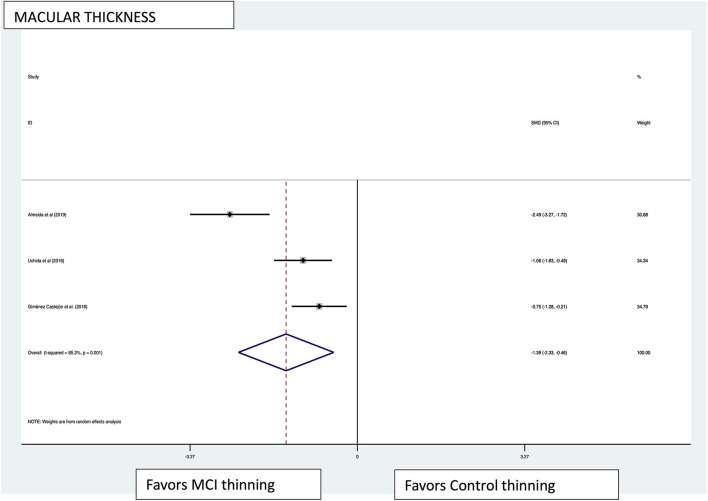
Macular thickness pooled effect size forest plot. Forest plot comparing the pooled effect size of change in macular thickness of Mild Cognitive Impairment (MCI) subjects and healthy controls. Weighted Hedges' g test for effect size analysis (blacksquare) and 95% confidence interval for each study.

**Figure 4:**
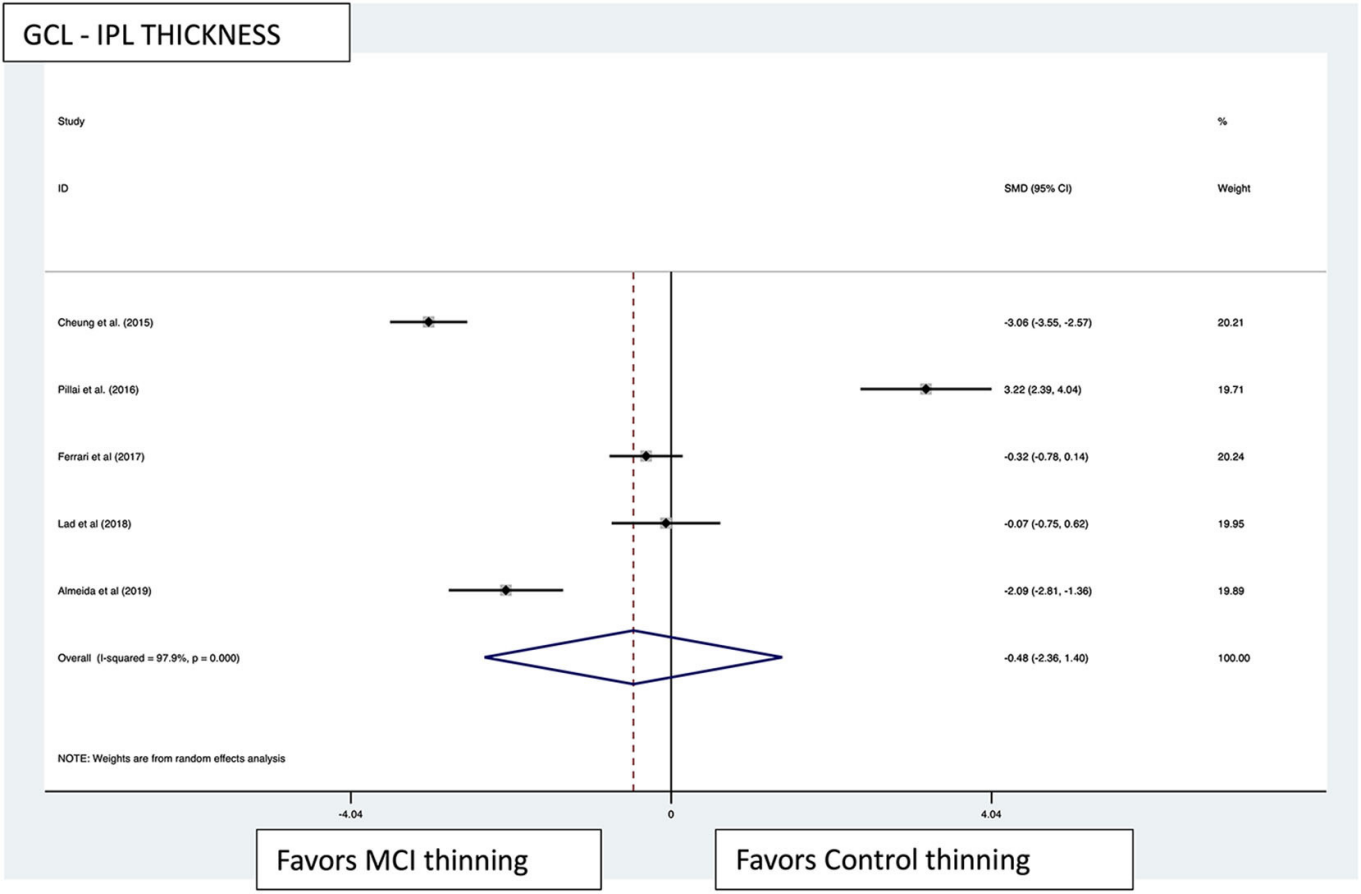
GCL-IPL thickness pooled effect size forest plot. Forest plot comparing the pooled effect size of change in ganglion cell layer-inner plexiform layer complex thickness (GCL-IPL) in Mild Cognitive Impairment (MCI) subjects and healthy controls. Weighted Hedges' g test for effect size analysis (black square) and 95% confidence interval for each study.

**Figure 5:**
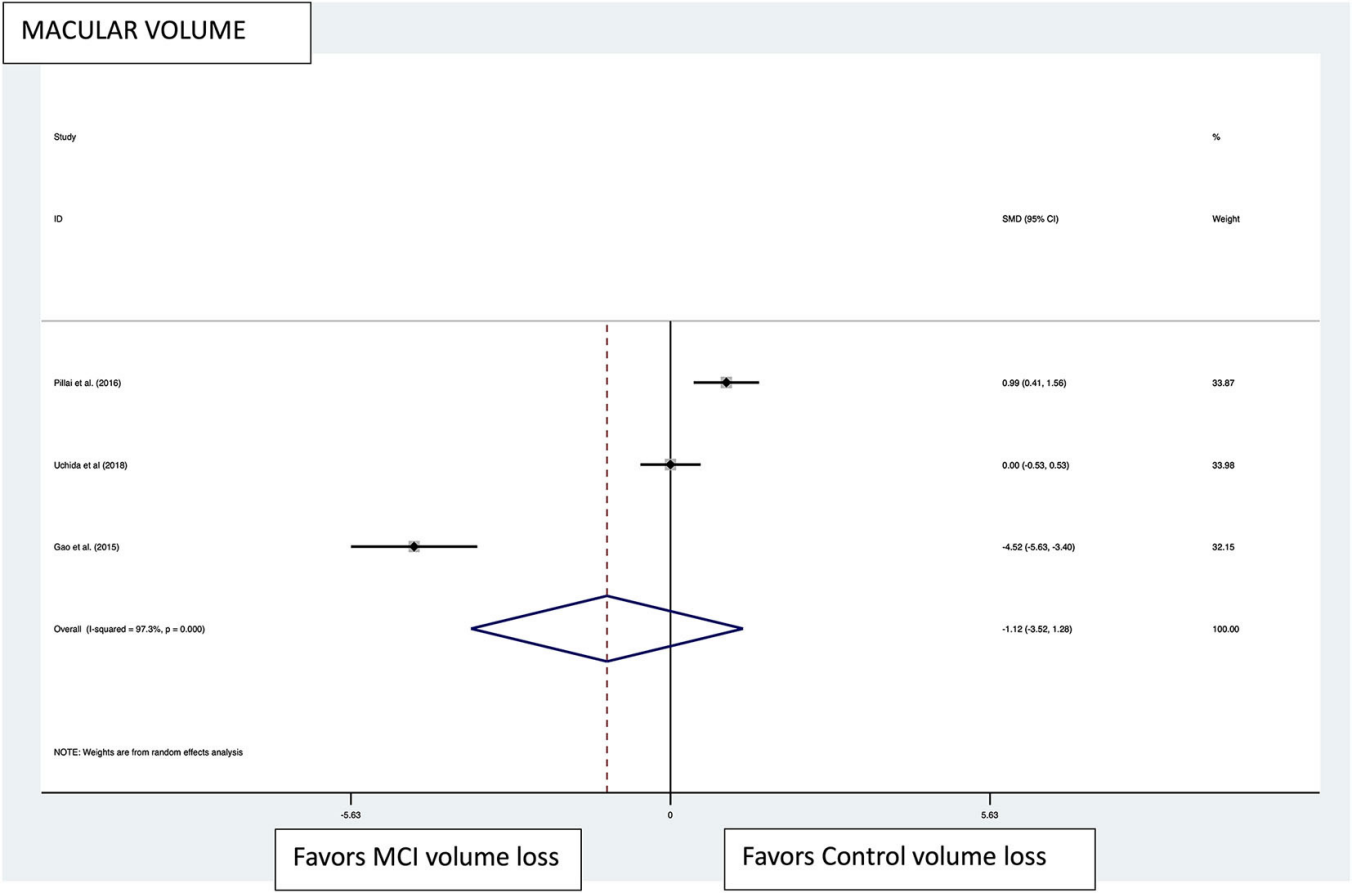
Macular volume pooled effect size forest plot. Forest plot comparing the pooled effect size of change in the macular volume between Mild Cognitive Impairment (MCI) subjects and healthy controls. Weighted Hedges' g test for effect size analysis (black square) and 95% confidence interval for each study.

**Figure 6:**
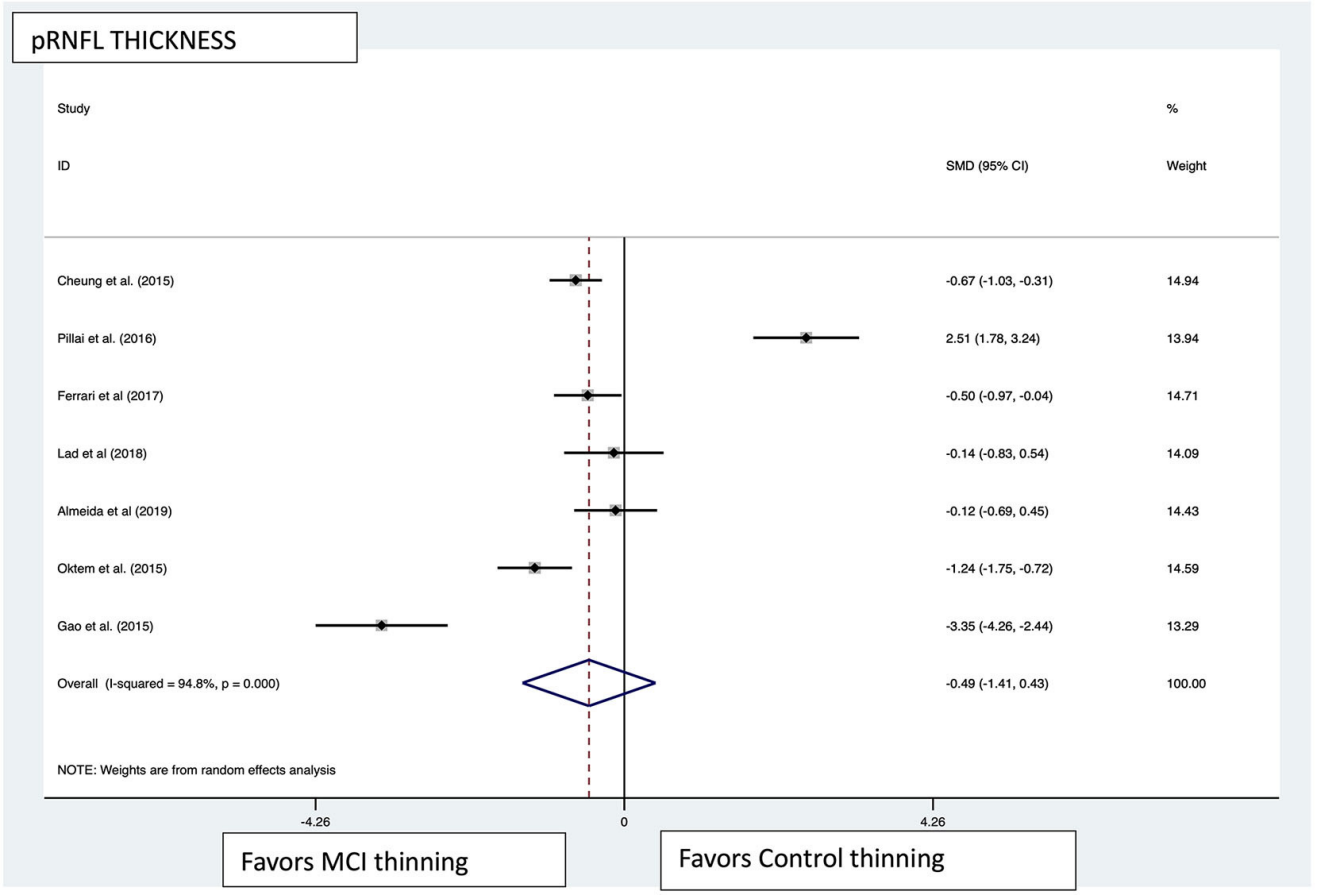
pRNFL pooled effect size forest plot. Forest plot comparing the pooled effect size of change in peripapillary retinal nerve fiber layer (pRNFL) thickness between Mild Cognitive Impairment (MCI) subjects and healthy controls. Weighted Hedges' g test for effect size analysis (blacksquare) and 95% confidence interval for each study.

Macular thickness pooled effect size analysis standard mean difference (or SMD) = −1.394 [−2.32, −0.46] with 2 degrees of freedom or *df* (*Z* = 2.93, *p* = 0.003). Heterogeneity: *I*^2^ = 85.3%, Chi-squared = 13.60, *p* = 0.001; GCL-IPL complex pooled effect size SMD was −0.47 [−2.36, 1.40] with 4 *df* (*Z* = 0.50, *p* = 0.619). Heterogeneity: *I*^2^ = 97.9%, Chi-squared = 192.60, *p* < 0.001; macular volume pooled effect size SMD = −1.119 [−3.51, 1.27] with 2 *df* (*Z* = 0.91, *p* = 0.360). Heterogeneity: *I*^2^ = 97.3%, Chi-squared = 74.17, *p* < 0.001; and pRNFL thickness pooled effect size SMD = −0.4 [−1.40, 0.43] with *df* (*Z* = 1.04, *p* = 0.298). Heterogeneity: *I*^2^ = 94.8%, Chi-squared = 114.37, *p* < 0.001. See Figures 3–6 for forest plots. Given the substantial heterogeneity for all variables, a multivariate meta-regression analysis was performed and did not reveal an explanation for the heterogeneity. The small number of studies contributes to the difficulty in interpreting the meta-analysis.

## Conclusions

We conducted a systematic review and meta-analysis of the literature for retinal OCT studies in the MCI stage that were published in the past 18 years, and 15 articles met the inclusion criteria. In examining the current literature, we identified significant variability between studies with regard to methodology and reporting. For instance, the exclusion criteria for confounding ocular diseases varied from study to study and many studies did not state how ocular pathology was excluded. In addition, the diagnostic criteria used for MCI were different, as discussed and as noted in Table 1. Older articles in this review used diagnostic criteria that were current at their respective publication time, however. As noted, the APOSTEL nomenclature was not used by manuscripts published earlier and making the methodology for OCT more difficult to compare between studies. Spectral-domain OCT (10 studies) technology was more commonly used compared with time-domain OCT technology (five studies). Although meta-analysis revealed large effect size for macular thickness, the heterogeneity for this variable and other retinal measures were substantial, but the reasons for the heterogeneity cannot be determined. Overall, the meta-analysis could mislead in the face of significant differences in cohort characterization, exclusion criteria, study design, OCT technology, and the number of studies.

Several important findings from this review may help to inform future work related to retinal OCT biomarkers for neurodegenerative diseases that present with mild cognitive impairment, specifically Alzheimer's disease. First, the studies reviewed offer evidence that differences in inner retinal measures and macular volume between MCI and healthy controls can be detected with OCT of the retina. Interestingly, studies that scanned both the macula and circumpapillary regions showed differences between MCI and controls in *either* the macula or the circumpapillary location but not both regions. The meta-analysis revealed significant heterogeneity between studies, and we believe that these findings suggest that retinal degeneration associated with the MCI stage is itself likely to be heterogeneous, with respect to the degree of degeneration between different regions of the retina and tissue involved (i.e., retinal ganglion cell, retinal ganglion cell axon, or optic nerve). Given what we know about the clinical and pathological heterogeneity of Alzheimer's disease, this is not surprising. Alternatively, the significant differences found in some studies could be spurious and related to the pitfalls of the small number of participants per study; confirmation of positive results awaits future studies with large cohorts defined by pathological biomarkers (i.e., CSF markers of tau and beta-amyloid) and the continued use of spectral-domain OCT, rather than time-domain OCT, will be important. At this time, the mechanism of retinal neuronal degeneration is unclear, and it remains uncertain whether the retinal ganglion cell (RGC) body, the RGC axons (i.e., the RNFL), and the optic nerve are each vulnerable to an initial insult with the other components of the neuron degenerating later in the course or whether each neuronal component is vulnerable to an insult at the same time in the disease course. The fact that there is not a clear pattern in studies that scanned both the macula and the pRNFL argues for the latter and for the importance of longitudinal studies that include scanning of the macula and the circumpapillary region in each participant. We also recommend including both eyes whenever possible, given the potential for differences between eyes, such as occurs between regions and sides of the brain in Alzheimer's disease.

It is important to note that studies using time domain OCT all showed significant differences between MCI and controls for circumpapillary RNFL measures. Starting in 2016, studies consistently used spectral-domain or swept-source OCT and automated segmentation, as well as the NIA-AA criteria for MCI, and these factors might have contributed to the lack of consistent positive findings in MCI vs. controls. Previously reported data regarding pRNFL differences between patients with Alzheimer's disease and healthy controls were also dependent on OCT technology (4). Spectral-domain OCT studies show an average of 4–12% difference in the pRNFL thickness between AD and controls, while studies that used time-domain OCT reported up to 40% difference between AD and controls (4, 17, 46). The differences in pRNFL measurements in studies using spectral-domain technology seem more consistent with the small, but significant, degree of retinal ganglion cell loss and RGC axonal degeneration and loss noted on histopathological studies of *post-mortem* AD retinas compared with control retinas (2). In the 15 studies reviewed in this article, those using spectral-domain technology showed that the difference in the total average pRNFL between MCI vs. controls was −2.6 μm (or approximately 1.8%), while the average difference was to −9.74 μm with time-domain OCT (or approximately 5.6%) (21, 23, 25, 26, 28–32, 34, 36–40). The reasons for these discrepancies between time domain and spectral domain remain unknown. The clinical significance of the difference in measurements between AD, CMI, and controls remains to be established. The changes in some pRNFL, for example, has been reported to be an 8–10 μm decrease for pRNFL in AD, compared with the 10–15 μm for pRNFL reported as the SD for healthy individuals in a recent meta-analysis (4). For MCI, the SD of pRNFL is 6.43 μm for the articles reviewed in this analysis.

The meta-analysis results demonstrated a large effect size in macular thickness favoring thinning in the MCI subjects compared with the healthy controls. Only three studies fulfilled the criteria for analysis, however. The *I*^2^ statistic demonstrated a substantial heterogeneity in this group of studies. No subgroup analysis was performed, as there were no defined subgroups in the studies. All other variables did not show statistically significant effect sizes in the analysis. A random effects meta-regression was performed, and given the parameters, it was not surprising that the *R*^2^ statistic and the *F* statistic were insignificant. The limited number of studies available for analysis and the highly heterogeneous results were factors in the lack of discovery of covariates that impacted effect size.

It is important to remember that MCI is a clinical construct that indicates a mildly abnormal cognitive profile, and the MCI designation alone does indicate whether there is AD pathological burden (i.e., amyloid (A), tau (T), or neurodegeneration (N). Given that recently published data from well-characterized cohorts that included amyloid biomarkers have not consistently shown differences in retinal OCT thickness measures between controls and AD, it is imperative that future studies use AD biomarkers, specifically A/T/N markers, to characterize participants (47, 48). It is understood that amyloid accumulation in Alzheimer's disease precedes the onset of cognitive impairment by 15–20 years, and there is evidence that clinical signs and symptoms of cognitive impairment are driven by tau and brain atrophy (i.e., neurodegeneration) and not amyloid alone. Only one of the 15 MCI studies reviewed here had biomarker data, and that study had a non-disease-specific measure of neurodegeneration that included volumetric MRI measures of the hippocampus and full brain (32). This study showed differences in volumetric MRI measures for MCI and AD patients vs. controls, but there were no significant differences in the pRNFL, GCL-IPL complex, or the macular cube volume at the group level, and the cohorts included AD, MCI, non-AD dementia, Parkinson's disease, and normal controls with the appropriate power to detect 12 μm differences with standard deviations of up to 15 μm (32). Although NIA-AA criteria for MCI and AD are more likely than not to have AD pathology, the clinical diagnosis cannot be a substitute for AD biomarkers.

Most importantly, we need to better understand the mechanism of retinal degeneration in AD. A feasible clinical approach is to study the relationship between retinal degeneration and well-characterized biomarkers of AD pathology including A/T/N biomarkers, cognitive dysfunction, as well as age at onset and white matter disease; longitudinal studies will be particularly important to generate hypotheses. Interestingly, a histopathological study demonstrated preferential loss of melanopsin retinal ganglion cells (mRGC) in retinas of people with Alzheimer's disease pathology (49), and mRGC account for 1–2% of the total retinal ganglion cell population (50). If Alzheimer's disease targets mRGC more than other types of retinal ganglion cells, then the minimal differences in retinal OCT measures between controls and those with clinical signs of Alzheimer's disease are not surprising.

Some studies have demonstrated the effect of beta-amyloid accumulation upon the capillary network of the retina, leading to vascular occlusion and subsequent degeneration (51, 52). The beta-amyloid accumulation has also been linked to a decrease in vascular endothelial growth factor in the macula (52). To date, only eight studies have used optical coherence tomography angiography (OCTA) to evaluate the changes of retinal vasculature in MCI subjects (53–60). Only two of them demonstrated a statistically significant difference in structures, specifically showing an enlargement of the foveal avascular zone, and a decrease of deep capillary plexus density in MCI subjects compared with controls (53, 59). Vascular changes may be associated with the pathophysiology of changes in the retinal structures, either by indirectly interfering with the measurements by OCT, or by directly decreasing vascular flow to those structures, and thus provoking degeneration, and further studies are needed to understand this particular mechanism since non-AD neurodegenerative disorders have been shown to have similar OCTA findings.

In summary, we advocate for following methodological approach for retinal OCT studies in Alzheimer's disease-related neurodegeneration: (1) the use of APOSTEL reporting, (2) spectral-domain OCT technology, (3) the use of the NIA-AA diagnostic criteria for healthy controls, MCI, and AD, (4) larger, longitudinal cohort studies or population-based studies, (5) OCT imaging of both eyes and scanning of the macula and circumpapillary regions to determine circumpapillary RNFL thickness, macular volume and thickness, and GCL-IPL complex thickness, and (6) AD biomarkers that include amyloid, tau, neurodegeneration markers along with other important associations (i.e., age at symptom onset, degree of white matter disease, and APOE 4 status). With a systematic approach, the question of whether OCT can be used as a biomarker for the MCI stage can be answered and will allow us to be closer to understanding the mechanisms of retinal degeneration in Alzheimer's disease and other neurodegenerative disorders.

## Data Availability Statement

The datasets presented in this study can be found in online repositories: http://doi.org/10.17632/8dxmc9xryb.2. The names of the repository/repositories and accession number(s) can be found in the article/supplementary material.

## Author Contributions

AM-V: conceptualization, methodology, investigation, formal analysis, data curation, writing, and visualization. PR-J: methodology, investigation, and formal analysis. VP: conceptualization, methodology, investigation, formal analysis, writing, review and editing, and supervision. All authors agree to be accountable for the content of the work.

## Conflict of Interest

The authors declare that the research was conducted in the absence of any commercial or financial relationships that could be construed as a potential conflict of interest.
